# Supplementary material for: Effects of yearling, juvenile and adult survival on reef manta ray (Manta alfredi) demography
Source: PeerJ. 2016 Aug 24;4:e2370. doi: 10.7717/peerj.2370 (PMC5012281; doi:10.7717/peerj.2370)
Supplement: Appendix S1 [file peerj-04-2370-s001.pdf]

APPENDIX

Effects of variations in yearling, juvenile and adult survival on reef manta ray (*Manta alfredi*) demography and population change

Isabel M. Smallegange[1], Isabelle B.C. van der Ouderaa[1] and Yara Tibiriçá[2]

[1]Institute for Biodiversity and Ecosystem Dynamics, University of Amsterdam, PO Box 94248, 1090 GE, Amsterdam, The Netherlands

[2]Association of Coastal Conservation of Mozambique, Praia de Zavora, s/n, Inharrime, Inhambane Province, Mozambique

Corresponding author:

Isabel Smallegange

Institute for Biodiversity and Ecosystem Dynamics, University of Amsterdam, PO Box 94248, 1090 GE, Amsterdam, The Netherlands

Email: i.smallegange@uva.nl

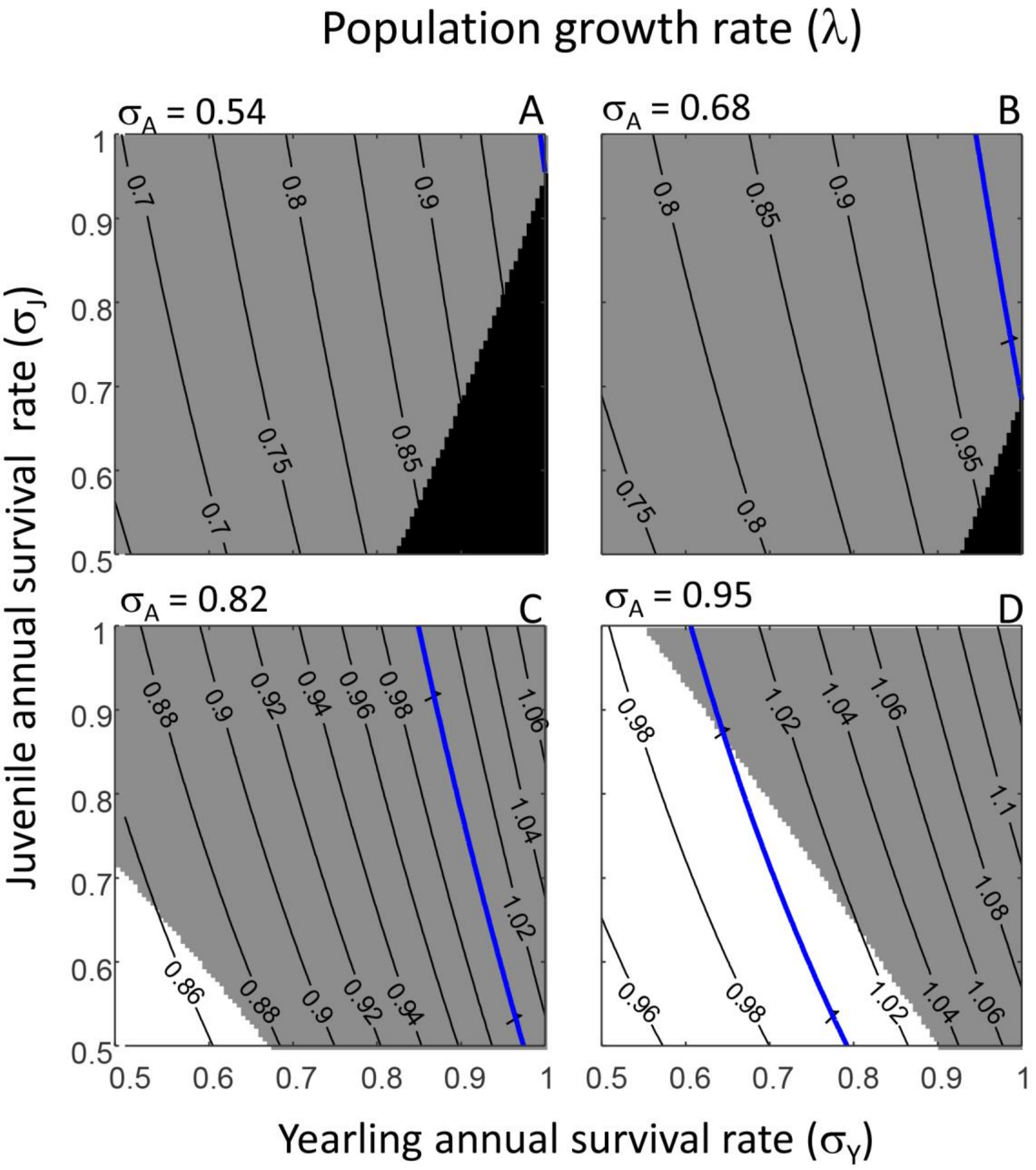

Population growth rate and sensitivity results. Predicted population growth rate λ in relation to yearling annual survival rate (σ_Y) and juvenile annual survival rate (σ_J) shown for each of four values of adult annual survival rate (σ_A): σ_A = 0.54 (80% of observed rate) (A); σ_A = 0.68 (observed rate) (B); σ_A = 0.82 (120% of observed rate) (C); and σ_A = 0.95 (140% of observed rate) (D). In each panel, isoclines denote equal values of the population growth rate λ. The blue

line in each panel denotes population stability at $\lambda = 1$; values higher than $\lambda = 1$ denote increasing populations and value lower than $\lambda = 1$ denote declining populations. The grey, black and white areas in panels denote the sensitivity results: white areas denote parameter combinations where the population growth rate is most sensitive to P_A, the rate at which adults survive and remain in the adult stage (Equation 3); grey areas denote parameter combinations where the population growth rate is most sensitive to G_J, the rate at which juveniles survive and grow into the adult life stage (Equation 3); black areas denote parameter combinations where the population growth rate is most sensitive to P_J, the rate at which juveniles survive and remain in the juvenile life stage (Equation 3).
